# Efficient Propranolol Delivery Enabled by Manganese‐Based Bioactive Metal–Organic Frameworks With High Loading Capacity

**DOI:** 10.1002/open.70319

**Published:** 2026-10-18

**Authors:** Hanieh Borhanipasvisheh, Soheil Deghani Siyahaki, Elham Gholamhosseinzadeh

**Affiliations:** ^1^ Department of Inorganic Chemistry Faculty of Chemistry University of Guilan Rasht Iran; ^2^ Chemical Engineering Department University of Kashan Kashan Iran; ^3^ Department of Chemistry University of Turin Turin Italy

**Keywords:** bio‐active MOFs, drug delivery, drug release kinetics, encapsulation, propranolol delivery

## Abstract

A green and efficient method was developed for propranolol delivery utilizing manganese‐based bioactive metal–organic frameworks (MOFs) as biocompatible drug carriers. Mn‐based bioactive MOFs with a high aspect ratio were synthesized and characterized for size (average, 17 µm), morphology, stability, elemental composition, crystallinity, functionality, charge character, and biocompatibility (a protein adsorption capacity of 0.03 µg µg^−1^ (5 h) and a hemolysis ratio of < 0.5%). Afterward, the MOFs were utilized for drug loading via an encapsulation method, and the drug release studies were also performed. The drug loading was confirmed using the zeta potential, CHN analysis, and thermogravimetric analysis (TGA) measurements. An encapsulation efficiency of 96% (0.5 W/W (drug/MOFs)) and a maximal capacity (*Q*_max_) of 0.526 mg mg^−1^ were obtained. The drug release showed that 93.72% of the loaded drug was released after 10 h for a composite of 0.25 W/W (drug/MOFs), while for 0.5 W/W (drug/MOFs), the drug release reached a plateau after 3 h. The kinetic studies showed longer release times and higher release rates for higher drug loading efficiency. Overall, herein, a bioactive platform for efficient delivery of propranolol was developed to improve the therapeutic efficacy by enhancing the drug loading and achieving controlled drug release.

## Graphical abstract

A green and efficient method has been developed for propranolol delivery utilizing manganese‐based bioactive MOFs as biocompatible drug carriers. Mn‐based bioactive MOFs with a high aspect ratio are synthesized and characterized for size, morphology, stability, elemental composition, crystallinity, functionality, charge character, and biocompatibility [a protein adsorption capacity of 0.03 µg µg^‐1^ (5 h) and a hemolysis ratio of <0.5%]. The MOFs are subsequently utilized for drug loading via an encapsulation method, and drug release studies are also performed.

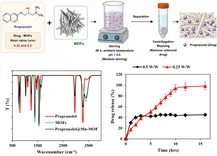

## 1 Introduction

Nanomaterials are known as a class of materials with nanoscale size (at least one dimension less than 100 nm) [1, 2, 3]. These nanoscale materials reveal unique characteristics, including distinct optical behavior [4, 5, 6, 7, 8, 9], unparalleled catalytic behavior [10, 11, 12, 13, 14, 15, 16, 17, 18, 19, 20], and different surface features [21, 22, 23, 24, 25, 26] compared to those of the bulk materials. Considering their great properties, the nanomaterials have been applied for different applications in sensing and detection [27, 28, 29, 30, 31, 32], catalysis [33, 34, 35, 36, 37, 38], tissue engineering [39, 40], nanodrug technology [41, 42, 43, 44, 45], drug delivery [43, 46, 47, 48, 49], energy technology [50], toxic material removal [51], enzyme immobilization [52], cancer treatment [53], and bioimaging [54]. Among the different applications, due to the high‐surface area and great reversible interaction ability of the nanomaterials with different molecules, these materials, especially metal–organic frameworks (MOFs), are considered ideal candidates for developing drug delivery systems.

Metal–organic frameworks (MOFs) are a class of porous materials formed by connecting inorganic cores with organic linkers to form extended (1D–3D) networks [55]. Currently, research on MOFs consists of two areas of interest, including the synthesis (design) and characterization of novel MOFs [56] and applications of previously synthesized MOFs [57].

Heretofore, different methods have been developed for the synthesis of various MOFs, for instance, solvothermal [58], nonsolvothermal [59], and direct precipitation reactions [60]. In the solvothermal methods, the synthesis process was performed in a closed vessel such as an autoclave, and the reaction temperature was higher than the boiling point of the reaction solvent [61]. Nonsolvothermal synthesis was carried out under simple conditions below or at the boiling point of the solvent [62]. However, a number of MOFs have been synthesized at room temperature [60]. The MOFs are suitable for a wide variety of applications in gas separation and gas storage [63], enzyme immobilization [60], constructing nanozyme‐based sensors [55], and drug delivery [64, 65, 66, 67, 68] due to many unique properties including tunable cavities, high porosity, and ultrahigh surface areas, as well as high peroxidase‐like activity of some MOFs [55]. Besides the well‐approved applications of MOFs in gas adsorption [69], sensing applications [70], and catalysis [71], many researchers are currently focused on the application of MOFs in safe drug delivery via designing biocompatible MOFs. For instance, in 2023, drug delivery on Mg‐MOF‐74 was studied by Pederneira et al., obtaining insights into the effect of drug solubility on pharmacokinetics [72]. Moreover, surface functionalization of Zr‐fumarate MOF was also reported for selective cytotoxicity and immune system compatibility in nanoscale drug delivery, which can be considered a great advantage of MOF‐based drug delivery relative to alternative systems [73]. In addition, in 2026, Salazar‐Muñoz et al. manufactured a drug delivery device based on MOF UiO‐66 for calcium‐channel‐blocker drugs [74].

Propranolol, a well‐known beta‐blocker drug, has been widely utilized for cardiovascular disorder treatment; however, the traditional oral forms of this drug are not able to sufficiently show its highest therapeutic efficacy because of a short half‐life of 3–6 h and low bioavailability. These limitations led to the use of the drug in repeated dosing throughout the day, causing drug concentration fluctuations in the blood and consequently reducing therapeutic efficacy [75]. Overcoming these challenges is possible by developing high‐throughput drug delivery systems to improve the drug loading capacity and achieve controlled drug release. As it is previously noted, among different applications of MOFs, their application as the highly powerful nanocarriers for drug delivery purposes is an interesting research field due to their green properties, high porosity, and ultrahigh surface areas [64, 65, 66, 67, 68].

Therefore, in this study, a high‐throughput green method was developed for propranolol delivery utilizing manganese‐based bioactive MOFs with high loading capacity as biocompatible and high‐throughput nanocarriers. In this regard, a Mn‐based bioactive MOF was designed and synthesized, providing needle‐like MOFs with a high aspect ratio, which are excellent candidates for drug loading. The as‐prepared MOFs were fully characterized. In addition, the zeta potential, CHN, and thermogravimetric analysis (TGA) analyses were used to confirm the drug loading onto the MOF. The biocompatibility of the MOFs was investigated by protein adsorption measurements. Afterward, the MOFs were utilized for the loading of the drug via the encapsulation method, and the drug release studies were also performed.

## 2 Experimental Section

### 2.1 Synthesis of the Bioactive MOFs

To synthesize the bioactive Mn–MOFs, briefly, 1.0 mmoL of MnCl_2_ (Merck Co.) was added to 40.0 mL of 50.0%v/v of MeOH/H_2_O solution, followed by addition of 2.0 mmoL of isopropylamine (Merck Co.) and 1.0 mmoL of terephthalic acid (Merck Co.) into the reaction mixture. The mixture was stirred for about 15.0 min at ambient temperature and then heated at 140 °C for about 24.0 h. Finally, the formed Mn–MOFs were collected by centrifugation, washed with 50.0% v/v of MeOH, and then dried under ambient conditions (Scheme 1).

**SCHEME 1:**
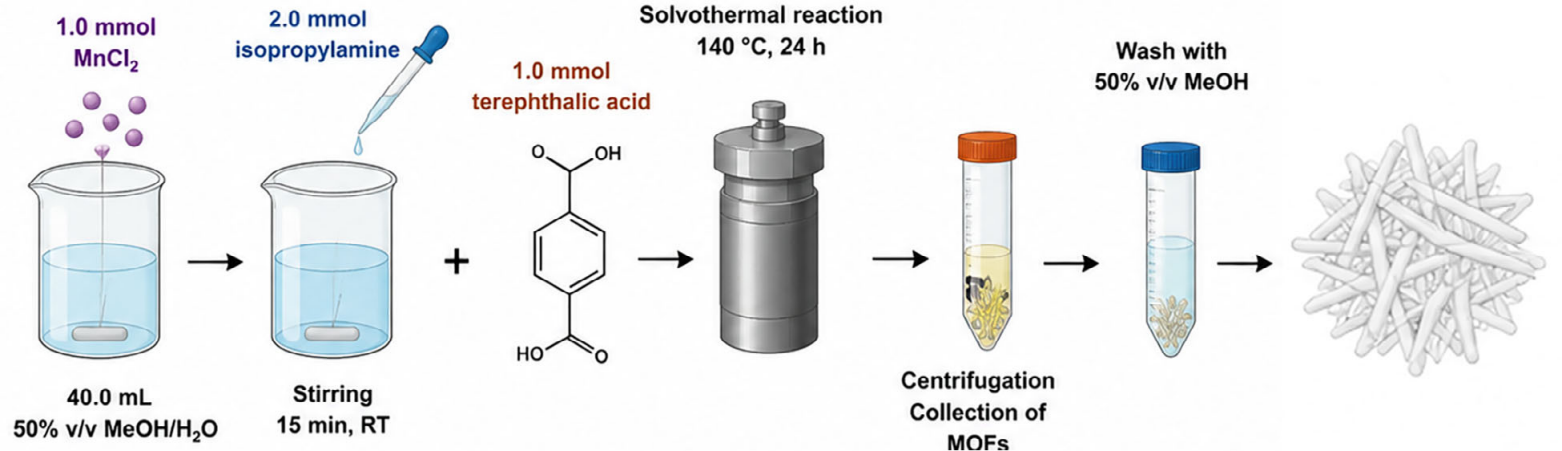
Schematic representation of the protocol of MOF synthesis.

### 2.2 Biocompatibility of Mn–MOFs

To do the protein adsorption tests, BSA (Merck Co.) was used as a model protein. In this regard, 1.0 mg of Mn–MOFs was suspended in phosphate buffer and incubated for about 24 h at ambient conditions; afterward, the MOFs suspension was then incubated at 37.0 °C (1 h) and then transferred to 1.0 mg mL^−1^ BSA solution. The MOF–BSA mixture was incubated at 37.0 °C (2 h) to complete the protein adsorption. After that, the MOF was collected, washed with water, and used for the protein desorption experiment. The process was done by incubating the MOFs at 37 °C with 2% SDS for about 1 h. The released BSA was quantified by direct UV–visible method via measuring the protein absorbance at 280.0 nm [76]. Thereafter, the protein adsorption capacity of the as‐prepared bioactive MOF was calculated by the following equation;

$$\text{Adsorbed protein}\left(\text{μg}\;{\text{μg}}^{-1}\right)=\frac{\text{initial BSA concentration}-\text{BSA concentration in the supernatant}}{\text{MOFs amount}}$$

### 2.3 Drug Loading and Release Tests

The as‐prepared MOFs were used for loading propranolol using the encapsulation method. To do this, different mass ratios of drug:MOFs (i.e., 0.25 and 0.5 W/W) were mixed for 48 h under medium stirring and ambient temperature at pH = 4.0. After that, the MOF@Drug composite was separated from the mixture, washed, and dried at room temperature. The amount of drug loaded onto the MOF carrier was then calculated. To test the drug release, the MOFs@drug composites (i.e., 0.25 and 0.5 W/W) were incubated in a dialysis membrane at pH 7.4 under medium stirring, and the amount of released drug into the solution was measured spectrophotometrically via sampling at different time intervals.

## 3 Results and Discussion

### 3.1 Characterization of Bioactive MOFs

The morphology of the synthesized bioactive MOF was evaluated by scanning electron microscopy (Figure 1a), revealing predominantly anisotropic needle‐like microcrystals with well‐defined elongated geometries, which is attributed to the directional crystal growth during the synthesis process. The observed anisotropic morphology indicated the preferential growth along a specific crystallographic direction, which is commonly observed in MOFs where strong metal–ligand coordination and ligand geometry promote one‐dimensional crystal extension, which are often associated with enhanced mass transport properties and can be considered a great advantage for applications in drug delivery by enhancing the drug loading efficiency. The size distribution of the bioactive MOF was also determined from the SEM images, providing a size (length) range of 5–40 µm with a mean size of about 17 µm (Figure 1b). Besides, the width of the MOF microcrystals was found to be 0.5–3 µm with a mean value of 1.5 µm (Figure 1c). The aspect ratio of the MOF as one of the most important characteristics that can define the loading capacity of the MOF was then calculated by dividing the MOF length to MOF width (L/W), showcasing a high aspect ratio over 3–15, while 40% of particles had a L/W value of 5–10 and about 30% of particles showed a higher L/W ratio of 10–15, confirming a broad but clearly anisotropic particle population, which can be associated with high loading capacity of the developed MOFs (Figure 1d).

**FIGURE 1:**
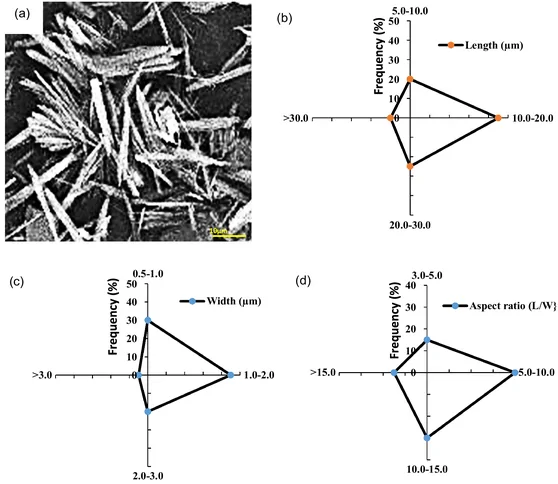
(a) SEM image, (b) size distribution as a function of particle length, (c) size distribution as a function of particle width, and (d) aspect ratio of the developed bioactive MOF.

The CHN elemental analysis was performed for the estimation of the organic content of the as‐prepared MOFs. Regarding the CHN elemental analysis, the as‐prepared MOFs have a total organic content of 78.30% (C = 72.49% and H = 4.48%). It should be mentioned that the 1.33% nitrogen in the bare MOFs almost certainly comes from the isopropylamine used in the synthesis. (Figure 2a).

**FIGURE 2:**
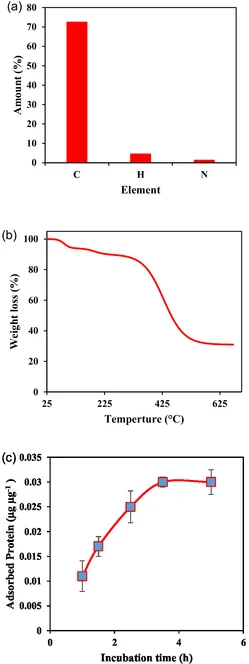
(a) CHN analysis, (b) TGA analysis, and (c) biocompatibility of the developed bioactive MOF.

The TGA analysis was finally utilized for evaluating the stability of the MOFs (Figure 2b); the results showed that the developed bioactive MOF exhibited high stability, retaining most of its mass up to approximately 400 °C before undergoing framework decomposition at higher temperatures.

The hydrolytic stability of the MOF was then evaluated by incubating 50.0 mg of the MOF with water, 1.0 M NaOH, and 1.0 M HCl for 24 h, and the mass loss of the MOFs was considered an index for assessing their hydrolytic stability. The results showed that the MOFs retained 98.2%, 97.5%, and 96.6% of their initial mass after 24 h of incubation with water, 1.0 M NaOH, and 1.0 M HCl, in order, proving their high hydrolytic stability.

### 3.2 Biocompatibility of the as‐Prepared MOFs

The hemocompatibility of the as‐prepared MOFs, an important issue, was checked to ensure their safety for drug delivery purposes. In this regard, initially, protein adsorption tests were performed on the as‐prepared MOFs. BSA as the presented protein in blood was utilized for the protein adsorption tests (Figure 2b), revealing that the bioactive MOFs adsorbed a protein amount as low as 0.011, 0.017, 0.025, 0.03, and 0.03 µg µg^−1^ at physiological pH (blood pH, 7.4) during MOF–protein interaction for about 1.0, 1.5, 2.5, 3.5, and 5 h, respectively (Figure 2c), confirming the high hemocompatibility of the as‐prepared MOFs which is the most important characteristic of a nanocarrier for drug delivery aims that primarily should be considered. Considering this fact that the protein is negatively charged at physiological pH, the very low protein adsorption capacity of the MOF is related to the high negative surface charge of the MOF (zeta potential of −18.5 mV), which can reduce the protein‐MOFs interactions and consequently the protein adsorption capacity of the as‐prepared MOF. To further evaluate the biocompatibility of the MOFs, their hemolysis ratio was evaluated; the results revealed that the hemolysis ratio of the as‐prepared MOFs was less than 0.5%, confirming their excellent biocompatibility. Moreover, the in vitro cytotoxicity of the MOFs and the MOFs@Drug was also evaluated using 100 µg mL^−1^ of them on HEK293 (Human Embryonic Kidney) cells (24 h). The results revealed a cell viability (%) of about 91.3% and 88.7% after 24 h for the MOFs and the MOFs@Drug, supporting their very good biocompatibility.

### 3.3 Drug Loading

Because propranolol is a weak base with a p*K*_a_ of approximately 9.4–9.5, it is nearly fully protonated (cationic) at pH of 4.0. This positive charge significantly influences its interaction with other surfaces and the stability of its formulations. On the other hand, in this pH range, the interactions between positively charged propranolol and negatively charged MOFs (zeta potential of −18.5 mV) are at their maximum value. To prove this assumption, the drug loading with a 0.5 W/W (drug:MOF) ratio was carried out at different pH values (Table 1); the results revealed that the drug loading increased by increasing pH from 2.0 to 4.0 and then decreased by further increasing pH. Therefore, to enhance the loading efficiency, the drug was loaded onto the MOFs at pH = 4.0. Regarding the drug loading, two different mass ratios of 0.25 and 0.5 W/W (drug:MOF) were tested (Scheme 2). The results are shown in Table 2, as shown in this table, the drug encapsulation efficiency was found to be as high as 96.0% and 90.8% for a mass ratio of 0.5 and 0.25 mg mg^−1^, in turn. Moreover, the MOFs show a very high capacity of about 0.48 mg mg^−1^ (i.e., loading 96% of the initial drug on the MOFs) along with a high drug loading efficiency of 48.0% for the 0.5 W/W ratio. However, the MOFs’ capacity for 0.25 W/W was calculated as 0. 227 mg mg^−1^ (i.e., loading 90% of the initial drug on MOFs) with a loading efficiency of 22.7%. These results indicated that both loading and encapsulation efficiencies, as well as the MOFs’ capacity, were improved by increasing the mass ratio of drug to MOFs and then leveled off. It should be mentioned that the mean drug loading capacity of the as‐synthesized MOFs was calculated to be as high as 0.353 mg mg^−1^_._

**TABLE 1:** The pH‐dependence drug loading results (Wdrug/WMOFs, 0.5).

pH	Loading, mg mg^−1^
2.0	0.430
4.0	0.480
5.5	0.396
7.4	0.251
9.0	0.106

**SCHEME 2:**
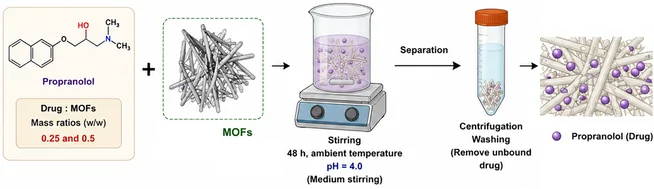
Schematic representation of the drug loading on the developed MOF.

**TABLE 2:** Drug loading results at pH = 4.0.

Wdrug/WMOFs, mg mg^−1^	MOFs capacity, mg mg^−1^	Encapsulation efficiency, %	Loading efficiency, %
0.5	0.480	96.0	48.0
0.25	0.227	90.8	22.7

To evaluate the loading behavior and determine the maximal loading capacity (*Q*_max_), the adsorption isotherms were constructed for drug loading. The drug concentration was calculated using the UV calibration curve by plotting its absorbance at 290 nm versus concentration (Figure S1). To determine the drug concentration in the supernatant, when the absorbance fell out of the calibration range, a dilution step was performed. The adsorption data are shown in Figure S2a, revealing that the drug loading increased with increasing drug concentration and finally reached a plateau, showcasing Langmuir behavior. To confirm this, the data were fitted using the linear form of the Langmuir adsorption isotherm (Figure S2b), providing a high *R* ^2^ value of 0.983 and a *Q*_max_ of 0.526 mg mg^−1^. Moreover, the Freundlich model was also applied to the experimental data, providing a poor *R* ^2^ value of 0.9185 (Figure S2c). Hence, the data confirmed a traditional Langmuir behavior for drug loading onto the developed MOFs.

### 3.4 Proving the Drug Loading Onto MOF

Initially, the zeta potential was used to confirm the successful loading of propranolol on the MOF (Figure 3a), revealing that the zeta value of the MOF decreased from −18.5 to −7.3 mV after propranolol, proving the loading of the drug on the bioactive MOF. Thereafter, the CHN analysis was also performed on the propranolol@MOF (Figure 3b), revealing C = 73.10%, H = 5.67%; propranolol@MOF for 0.48 mg mg^−1^ loading. Moreover, the nitrogen content (chemical formula of drug = C_16_H_21_NO_2_) of the propranolol@MOF was found to be 2.67%, which was higher than that of the bare MOFs, indicating the presence of propranolol on the MOFs. Based on the above experiments, it can be concluded that the lidocaine was successfully loaded onto the as‐prepared MOFs. Besides, the total organic content (Figure 3c) of the MOFs was increased from 78.30% (bare MOFs) to 81.44% revealing successful loading of the drug on the MOF. Finally, the TGA analysis further confirmed the encapsulation of propranolol within the framework (Figure 3d). For the propranolol‐loaded MOF (Propranolol@MOF), three distinct weight‐loss regions were observed in the TGA pattern, including 5.2% weight loss at 30–120 °C, attributed to the removal of physically adsorbed water molecules and residual solvent trapped within the pores of the framework, and secondary weight loss of about 46.5% over 200–350 °C, corresponding to the thermal decomposition of encapsulated propranolol. Based on this decomposition event, the drug loading was estimated to be about 47 wt%, which is in good agreement with the drug loading results. These findings are consistent with efficient drug encapsulation and demonstrate that the Mn–MOF can accommodate a significant amount of propranolol while maintaining structural integrity under physiological temperature conditions.

**FIGURE 3:**
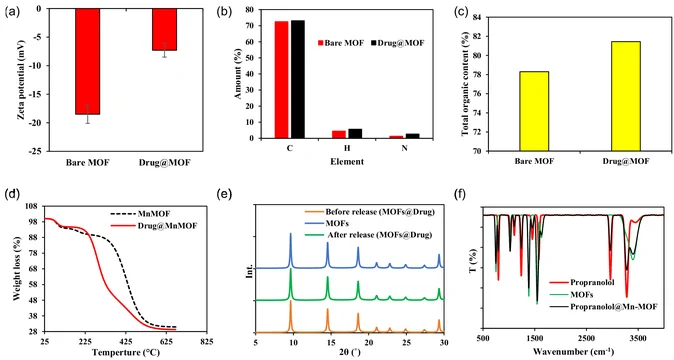
Proving the drug loading on MOF: (a) Zeta potential results, (b) CHN results, and (c) total organic contents, (d) TGA results before and after drug loading, (e) XRD patterns, and (f) FT‐IR spectra.

Moreover, PXRD patterns of the MOFs and MOFs@drug (before and after drug release experiments) were recorded to evaluate phase identity and drug incorporation into the MOFs (Figure 3e). The pristine material exhibited the characteristic crystalline peaks of the Mn–MOFs at 2*θ* of 9.62°, 14.58°, 18.64°, 21.24°, 22.9°, 25.0°, 27.52°, and 29.3°, confirming the high crystallinity of the as‐prepared MOFs. The main MOFs XRD peaks were preserved after propranolol loading and after drug release experiments in PBS (pH 7.4), although changes in relative intensity and peak breadth were observed, proving the retention of the crystalline features of the MOFs under drug loading and release conditions.

Moreover, the FTIR spectra of the MOFs, propranolol, and the MOFs@drug were recorded to assess the coexistence of the framework carboxylate bands and propranolol‐associated functional groups (Figure 3f). In the propranolol FT‐IR spectrum, the peaks at 3280.3, 2964.5, 1579.6, 1240.1, 1030.4, and 798.3 cm^−1^ correspond to the N─H/O─H stretching, C─H stretching, aryl C═C stretching, aryl O─CH_2_ asymmetric stretch, aryl O─CH_2_ symmetric stretch, and α‐substituted naphthalene, in order. Regarding the MOFs spectra, the peaks at 3400.1, 1546.01, 1386.28, 751.51, 1533.2, and 1020.2 cm^−1^ are attributed to the O─H stretching of water, *ν*_as_(COO^−^), ν_s_(COO^−^), Mn–terephthalate coordination‐associated band, and *ν*_as_(COO^−^), respectively. After drug loading, the FT‐IR spectrum revealed the characteristic bands of both MOFs and the drug with changes in the intensity or shifts in the peak positions, which can be related to the microenvironmental changes after drug loading.

The BET analysis was performed before and after drug loading (Table S1), revealing a BET surface area of 77.2 m^2^ g^−1^, a pore volume of 0.082 cm^3^ g^−1^, and a mean pore diameter of 3.7 nm for the developed MOFs. After drug loading, a BET surface area of 25.3 m^2^ g^−1^, a pore volume of 0.027 cm^3^ g^−1^, and a mean pore diameter of 1.5 nm were observed, revealing successful drug loading.

### 3.5 Drug Release Experiments and Kinetics Studies

Because propranolol is a well‐known beta‐blocker drug that has been widely utilized for cardiovascular disorder treatment, to achieve adequate treatment performance, the loaded drug should be able to be released at an excellent rate under physiological conditions. Hence, its release under physiological conditions (pH = 7.4) using a dialysis membrane in phosphate buffer was investigated (Scheme 3). Concerning drug release, the results were recorded over 0.0–16.0 h, and the plot of release efficiency as a function of release time was constructed for both mass ratios (drug: MOFs) of 0.25 and 0.5 W/W (Figure 4a); as shown, the drug release reached 42.0% after 3 h for the mass ratio of 0.5 W/W and then leveled off. In contrast, the release percentage reached 50% after 5 h and 93.72% after 10 h for the 0.25 W/W (drug/MOFs) and then leveled off. As shown in Figure 4, with increasing the drug loading percentage (0.5 W/W), the release time was prolonged to reach the plateau, and it is speculated that longer release times and higher release rates could be achieved by increasing the drug loading amount. This can be proved by considering the kinetics of the drug release throughout the dialysis membrane. The amount of Mn(II) leached to the medium was finally determined using inductively coupled plasma optical emission spectroscopy (ICP‐OES), revealing about 0.63 µg L^−1^ after 16 h for the 0.5 W/W ratio, suggesting negligible leaching amounts. To conduct the kinetics investigations, the Higuchi kinetic model, one of the most popular kinetic models, was applied to represent the experimental data. To construct the Higuchi kinetic plot, the curve of drug release percentage (%) as a function of time^1/2^ was provided (Figure 4b). As shown in Figure 4b, the corresponding Higuchi kinetic plot showed a linear relationship between drug release and time^1/2^ at 0.25 W/W, while a nonlinear behavior was obtained for 0.5 W/W, showing longer release times and higher release rates for higher drug loading efficiency. Moreover, the first‐order kinetics of the drug release were evaluated by plotting the log(% remaining drug) versus the release time (Figure 4c). The results provide a release function of *y* = −0.0101x + 1.8466 and a very poor *R* ^2^ of 0.4085 for 0.5 W/W drug:MOF ratio, indicating that for high drug:MOF ratios, the first‐order kinetics are not able to describe the drug release behavior. The Higuchi kinetic model provided a higher *R* ^2^ of 0.5938 and a drug release equation of *y* = 3.7491x + 32.777 (see Figure 4b), showing a nonlinear behavior of the model. Hence, the nonlinear fit. In contrast, at low ratios (0.25 W/W), both models showed very good ability to describe the drug release with linear release behavior; however, the Higuchi kinetic model (*y* = 34.576x–21.537; *R* ^2^ = 0.9823) was found to be able to describe the dataset more accurately than the first‐order kinetic model (*y* = −0.1189x + 2.1526; *R* ^2^ = 0.938). Finally, the Korsmeyer–Peppas model was plotted for both ratios; the results are shown in Figure 4d, revealing an equation of *y* = 0.1595x−0.3772 and an *R* ^2^ of 0.6838 for 0.5 W/W, while an equation of *y* = 0.8929x−2.2931 with an *R* ^2^ of 0.9745 for 0.25 W/W was provided. According to the *R* ^2^ values for all models, the Korsmeyer–Peppas model showed a higher ability to describe drug release behavior at a 0.5 W/W ratio, while the Higuchi kinetic model was the best model to describe the drug release behavior at a 0.5 W/W ratio.

**SCHEME 3:**
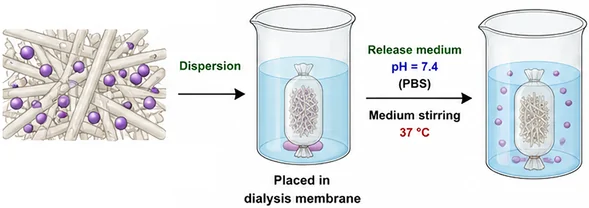
Schematic representation of the drug release from the developed drug‐loaded MOF.

**FIGURE 4:**
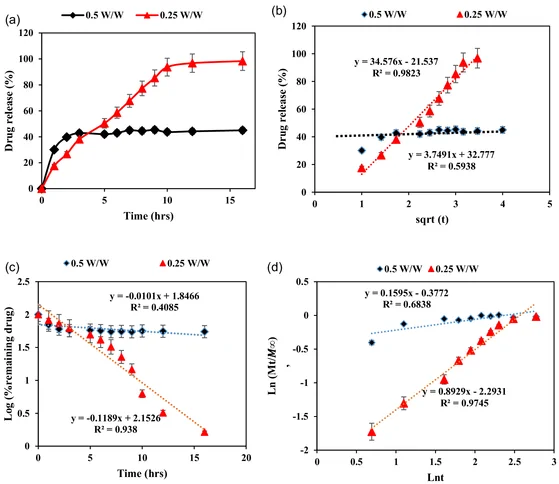
Drug release results: (a) plots of release efficiency versus release time, (b) Higuchi kinetic model, (c) the first‐order kinetics of the drug release, and (d) Korsmeyer–Peppas kinetic model.

Drug release experiments at pH = 5.5 for 0.25 W/W (MOFs/Drug) ratio were then performed to further evaluate the drug release behavior (Figure S3). The results revealed that the release percentage reached 60% after 2 h and 92.5% after 8 h for the 0.25 W/W (drug/MOFs) ratio and then leveled off. These results demonstrated that the drug release at physiological pH was more effective than at an acidic pH.

Besides, as a control experiment, the drug release experiments for the free drug were also performed at pH = 7.4 (Figure S4), revealing a drug release percentage of 72.8% after 0.5 h and 95.7% after 2 h, revealing very fast and uncontrolled drug release.

## 4 Conclusion

A high‐throughput green method was developed for propranolol delivery utilizing manganese‐based bioactive MOFs with high loading capacity as biocompatible and high‐throughput drug carriers. In this regard, a Mn‐based bioactive MOF was designed and synthesized, providing needle‐like MOFs with a high aspect ratio, which are excellent candidates for drug loading. The as‐prepared MOFs were fully characterized for size, morphology, stability, elemental composition, charge character, and biocompatibility. Afterward, the MOFs were utilized for the loading of the drug via the encapsulation method, and the drug release studies were also performed. The drug loading was confirmed using the zeta potential, CHN analysis, and TGA measurements. An encapsulation efficiency of 96% with a drug loading as high as 0.48 mg mg^−1^ was provided for the developed MOFs. The drug release showed that 93.72% of the loaded drug was released after 10 h for a composite of 0.25 W/W (drug/MOFs), while for 0.5 W/W (drug/MOFs), the drug release reached a plateau after 3 h. Besides, the kinetic studies showed longer release times and higher release rates for higher drug loading efficiency. Overall, this study presents a high‐throughput bioactive platform for efficient delivery of propranolol to improve therapeutic efficacy by enhancing drug loading and achieving controlled drug release. Despite the great advantages of this protocol, including biocompatibility, high loading efficiency, and excellent controlled drug release rate, the study included in vitro studies. As a future perspective on this study, comprehensive in vivo drug delivery studies on the developed green protocol can be performed.

## Conflicts of Interest

The authors declare no conflicts of interest.

## Supporting information

Supplementary Material
