# Supplementary material for: Efficient Propranolol Delivery Enabled by Manganese‐Based Bioactive Metal–Organic Frameworks With High Loading Capacity
Source: ChemistryOpen. 2026 Oct 18;15(11):e70319. doi: 10.1002/open.70319 (PMC13677820; doi:10.1002/open.70319)
Supplement: Supplementary file 1 [file OPEN-15-e70319-s001.pdf]

Supporting information

Efficient Propranolol Delivery Enabled by Manganese-based Bioactive MOFs with High Loading Capacity

Hanieh Borhanipasvisheh[1,*], Soheil Deghani Siyahaki[2], Elham Gholamhosseinzadeh[3]

[1]Department of Inorganic Chemistry, Faculty of Chemistry, University of Guilan, Rasht, Iran

[2]Chemical Engineering Department, University of Kashan, Kashan, Iran

[3]Department of Chemistry, University of Turin, Turin, Italy

** **Email:**haniehborhanipasisheh@gmail.com*

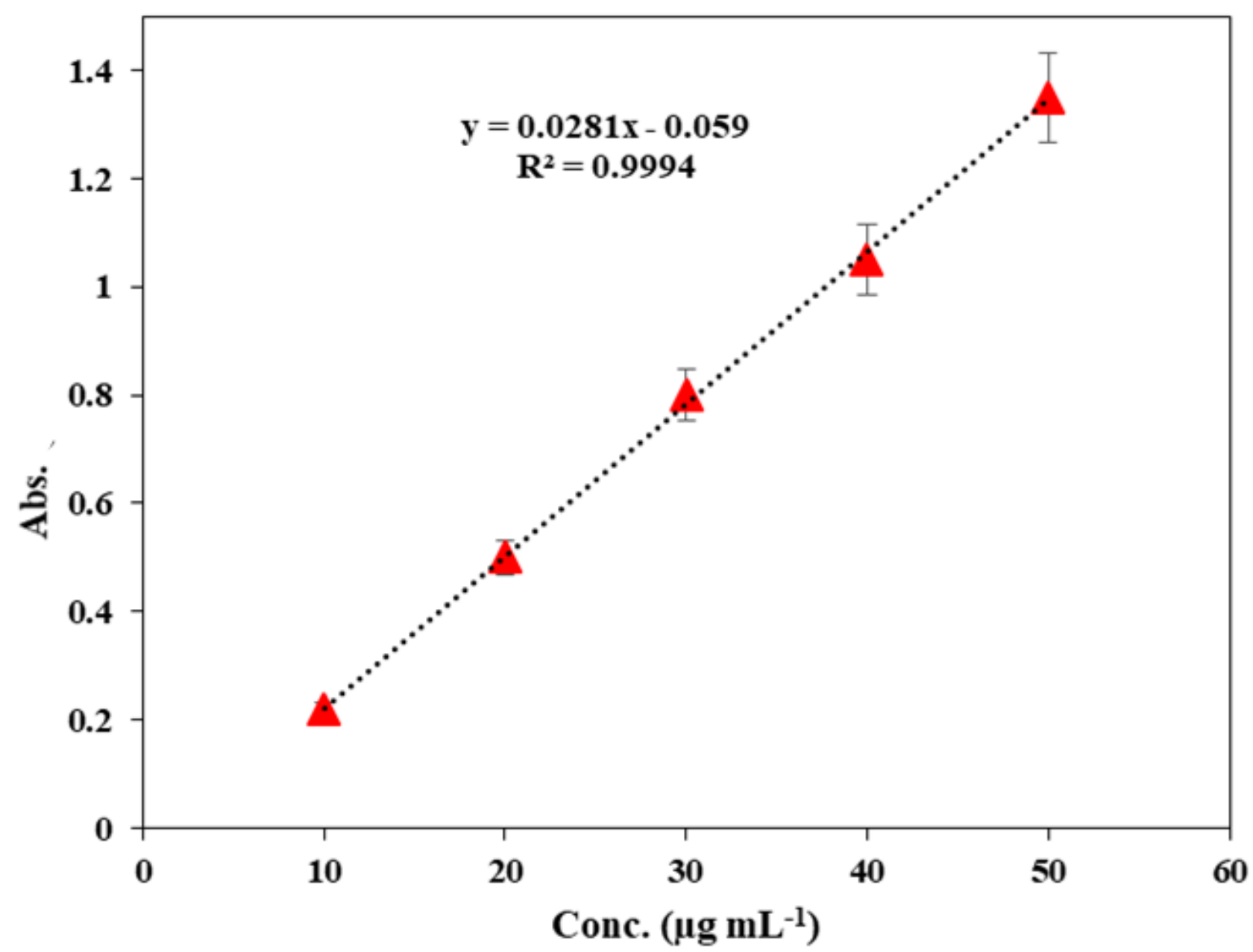

Figure S1. Calibration curve for propranolol quantification.

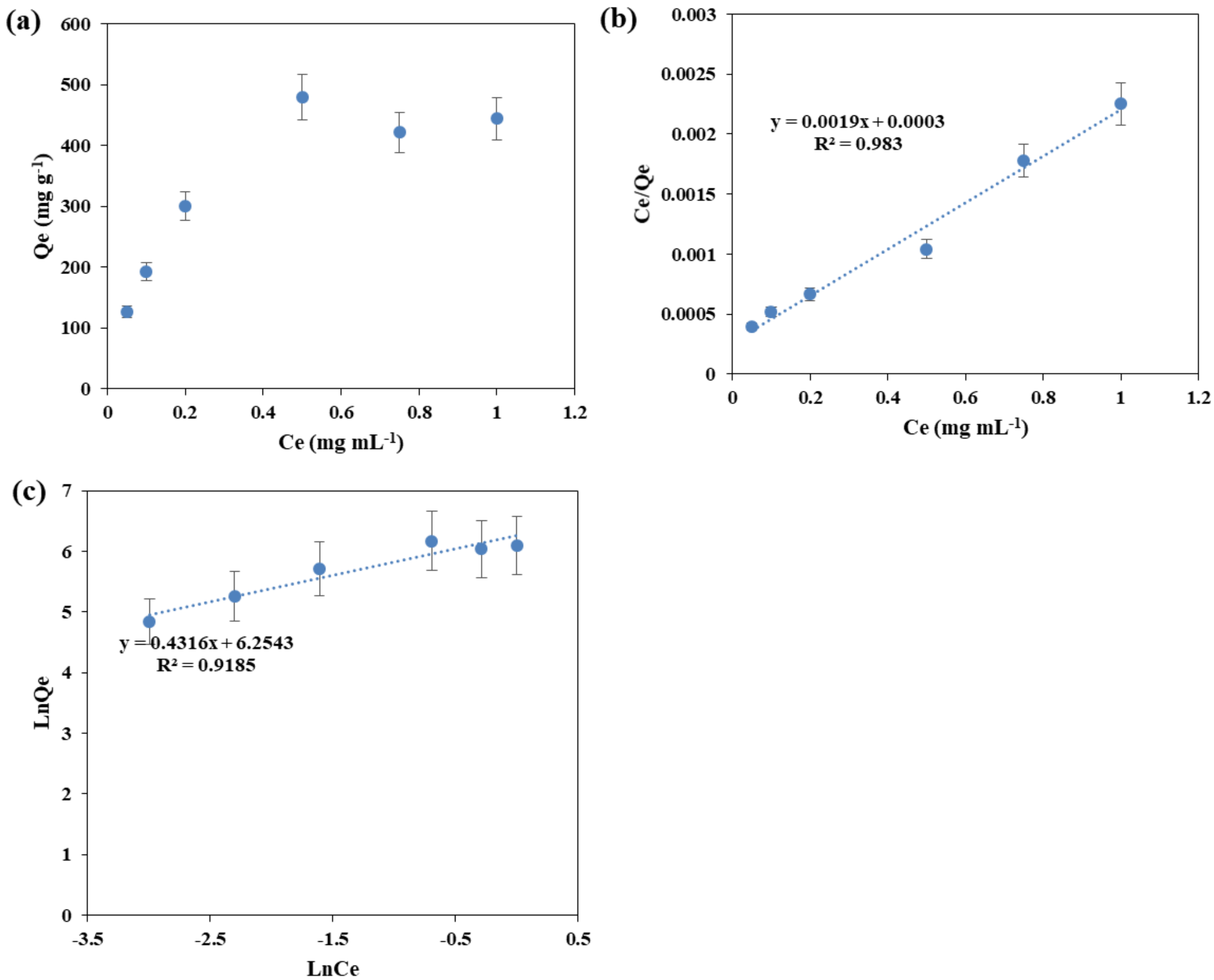

Figure S2. (a) Q_e *vs.* C_e curve, (b) Langmuir adsorption isotherm, and (c) Freundlich adsorption model for drug loading onto the developed MOFs.

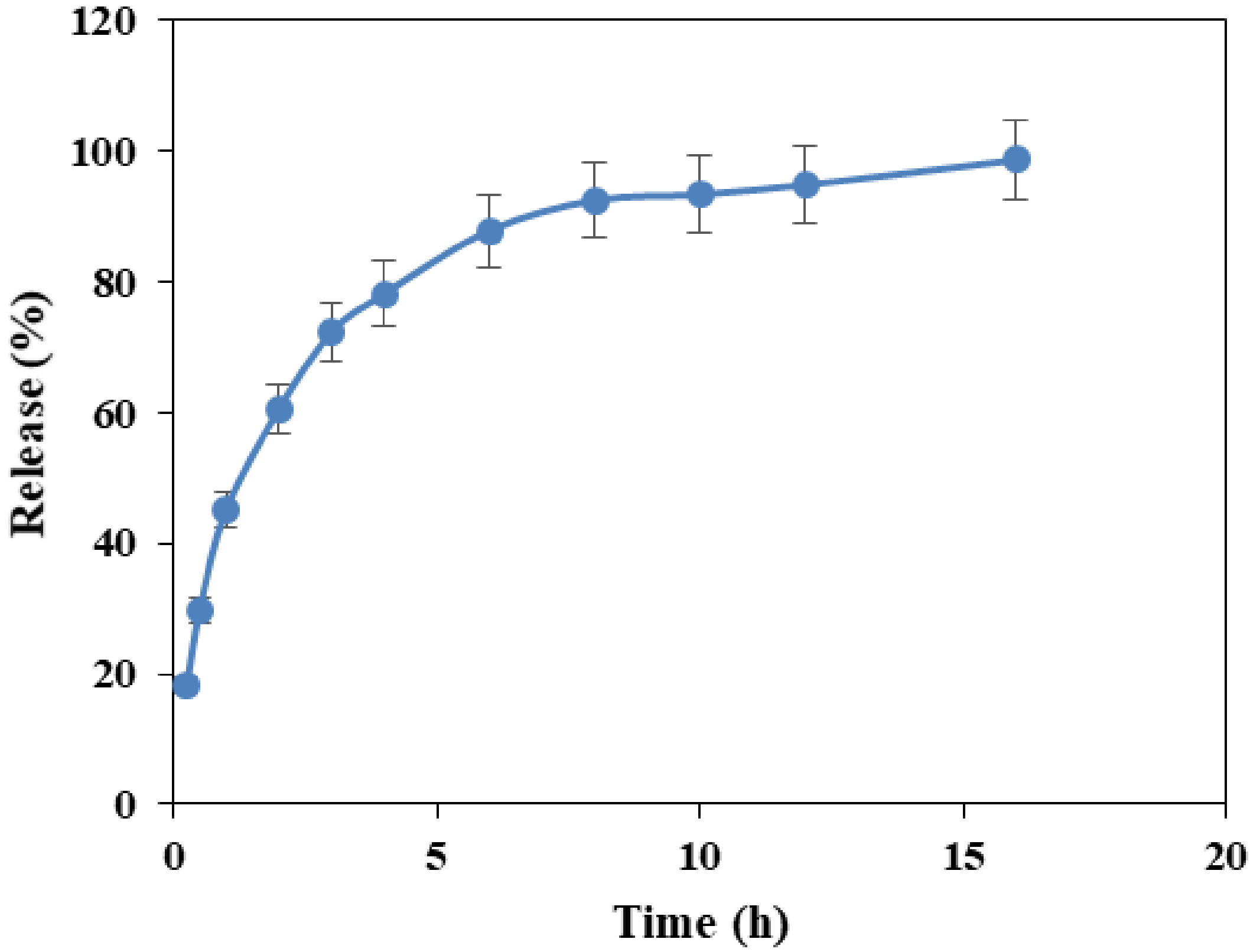

Figure S3. Drug release experiments at pH=5.5 for 0.25 W/W (MOFs/ Drug) ratio.

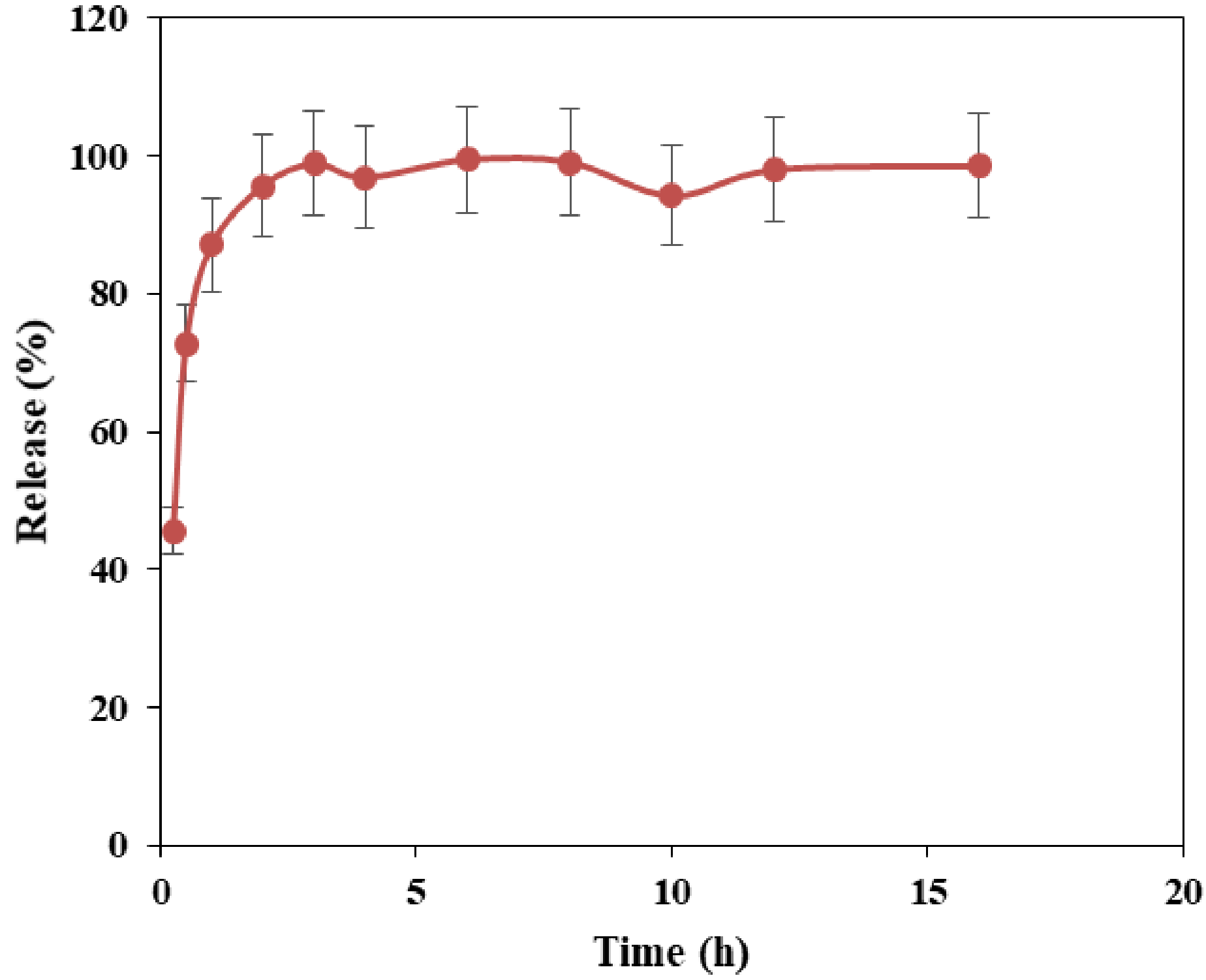

Figure S4. Drug release experiments for the free drug at pH=7.4

Table S1. BET results before and after drug loading.

Material	Surface area (m^2 g^{-1})	Pore volume (cm^3 g^{-1})	Mean pore diameter (nm)
MOFs	77.2	0.082	3.7
MOFs@Drug	25.3	0.027	1.5
